# Diversity of the type VI secretion systems in the *Neisseria* spp

**DOI:** 10.1099/mgen.0.000986

**Published:** 2023-04-13

**Authors:** Alan Calder, Lori A.S. Snyder

**Affiliations:** ^1^​ School of Life Sciences, Pharmacy, and Chemistry, Kingston University, Penrhyn Road, Kingston upon Thames, KT1 2EE, UK

**Keywords:** Type 6 secretion system, Type VI secretion system, T6SS, Toxin antitoxin system, *Neisseria* spp, Commensal *Neisseria*

## Abstract

Complete Type VI Secretion Systems were identified in the genome sequence data of *Neisseria subflava* isolates sourced from throat swabs of human volunteers. The previous report was the first to describe two complete Type VI Secretion Systems in these isolates, both of which were distinct in terms of their gene organization and sequence homology. Since publication of the first report, Type VI Secretion System subtypes have been identified in *Neisseria* spp. The characteristics of each type in *N. subflava* are further investigated here and in the context of the other *Neisseria* spp., including identification of the lineages containing the different types and subtypes. Type VI Secretion Systems use VgrG for delivery of toxin effector proteins; several copies of *vgrG* and associated effector / immunity pairs are present in *Neisseria* spp. Based on sequence similarity between strains and species, these core Type VI Secretion System genes, *vgrG*, and effector / immunity genes may diversify via horizontal gene transfer, an instrument for gene acquisition and repair in *Neisseria* spp.

## Data Summary

Details for the PubMLST commensal *Neisseria* spp isolates can be found in (Table S1, available with the online version of this article). Sequence data for commensal *Neisseria* spp. investigated are available in GenBank under the following accession numbers: *Neisseria subflava* strains M18660 (NZ_CP031251; url - https://www.ncbi.nlm.nih.gov/nuccore/NZ_CP031251.1); C2011020198 (NZ_POXM00000000; url – https://www.ncbi.nlm.nih.gov/nuccore/NZ_POXM00000000.1); C2011009653 (NZ_POXL00000000; url – https://www.ncbi.nlm.nih.gov/nuccore/NZ_POXL00000000.1); ATCC 49275 (NZ_CP039887; url - https://www.ncbi.nlm.nih.gov/nuccore/NZ_CP039887.1); NJ9703 (NZ_ACEO00000000; url - https://www.ncbi.nlm.nih.gov/nuccore/NZ_ACEO00000000.2); C2005001510 (NZ_POWU00000000; url –https://www.ncbi.nlm.nih.gov/nuccore/NZ_POWU00000000.1); C2009010520 (NZ_POXD00000000; url –https://www.ncbi.nlm.nih.gov/nuccore/NZ_POXD00000000.1); C2011004960 (NZ_POXK00000000; url –https://www.ncbi.nlm.nih.gov/nuccore/NZ_POXK00000000.1); *Neisseria perflava* strains UMB0023 (NZ_PKJQ00000000; url - https://www.ncbi.nlm.nih.gov/nuccore/NZ_PKJQ00000000.1); UMB0210 (NZ_PKJP01000000; url – https://www.ncbi.nlm.nih.gov/nuccore/NZ_PKJP00000000.1); *Neisseria flavescens* strains CDNF2 (NZ_LAEI01000000; url –https://www.ncbi.nlm.nih.gov/nuccore/NZ_LAEI00000000.1); N13 (NZ_CAABZZ010000000; url – https://www.ncbi.nlm.nih.gov/nuccore/NZ_CAABZZ000000000.1); N57 (NZ_CAACAD010000000; url – https://www.ncbi.nlm.nih.gov/nuccore/NZ_CAACAD000000000.1); *Neisseria elongata* strains M15910 (NZ_CP031255; url – https://www.ncbi.nlm.nih.gov/nuccore/NZ_CP031255); C2013010062 (NZ_POXS01000000; url –https://www.ncbi.nlm.nih.gov/nuccore/NZ_POXS00000000.1); C2010010207 (NZ_POXH01000000; url – https://www.ncbi.nlm.nih.gov/nuccore/NZ_POXH00000000.1); 404_NMEN (NZ_JVIM01000000; url – https://www.ncbi.nlm.nih.gov/nuccore/NZ_JVIM00000000.1); 431_NMEN (NZ_JVHH01000000; url – https://www.ncbi.nlm.nih.gov/nuccore/NZ_JVHH00000000.1); *N. mucosa* strains FDAARGOS_260 (NZ_CP020452; url –https://www.ncbi.nlm.nih.gov/nuccore/NZ_CP020452); ATCC 19696 (CP028150; url –https://www.ncbi.nlm.nih.gov/nuccore/CP028150); CCH7 A10 (NZ_LSIR01000000; url – https://www.ncbi.nlm.nih.gov/nuccore/NZ_LSIR00000000.1); NCTC 10774 (NZ_UGRT01000000; url – https://www.ncbi.nlm.nih.gov/nuccore/NZ_UGRT00000000.1); ATCC 25996 (NZ_ACDX02000000; url – https://www.ncbi.nlm.nih.gov/nuccore/NZ_ACDX00000000.2); N32 (NZ_CAACAC010000000; url – https://www.ncbi.nlm.nih.gov/nuccore/NZ_CAACAC000000000.1); C2004002444 (NZ_POWT01000000; url – https://www.ncbi.nlm.nih.gov/nuccore/NZ_POWT00000000.1); C2008000159 (NZ_POWX01000000; url - https://www.ncbi.nlm.nih.gov/nuccore/NZ_POWX00000000.1); *Neisseria sicca* strains ATCC 29256 (NZ_ACKO02000000; url - https://www.ncbi.nlm.nih.gov/nuccore/NZ_ACKO00000000); DSM 17713 (NZ_CP059566; url -https://www.ncbi.nlm.nih.gov/nuccore/NZ_CP059566); C2010005502 (NZ_POXG01000000; url - https://www.ncbi.nlm.nih.gov/nuccore/NZ_POXG00000000.1); UMB0321 (NZ_PKJO01000000; url - https://www.ncbi.nlm.nih.gov/nuccore/NZ_PKJO00000000.1); VK64 (NZ_AJMT00000000; url - https://www.ncbi.nlm.nih.gov/nuccore/NZ_AJMT00000000.1); C2014002478 (NZ_POXX00000000; url - https://www.ncbi.nlm.nih.gov/nuccore/NZ_POXX00000000.1); DE0496 (NZ_VDQK00000000; url-https://www.ncbi.nlm.nih.gov/nuccore/NZ_VDQK00000000.1); DE0493 (NZ_VDQM00000000; url-https://www.ncbi.nlm.nih.gov/nuccore/NZ_VDQM00000000.1); DE0367 (NZ_VDZJ00000000; url-https://www.ncbi.nlm.nih.gov/nuccore/NZ_VDZJ00000000.1); *N. macacae* strains MGYG-HGUT-01381 (NZ_CABKQF000000000; url - https://www.ncbi.nlm.nih.gov/nuccore/NZ_CABKQF000000000); ATCC 33926 (AFQE01000000; url - https://www.ncbi.nlm.nih.gov/nuccore/AFQE00000000.1); *Neisseria bergeri* strains C2008000328 (NZ_POWY00000000; url - https://www.ncbi.nlm.nih.gov/nuccore/NZ_POWY00000000.1); C2008000329 (NZ_POWZ00000000; url - https://www.ncbi.nlm.nih.gov/nuccore/NZ_POWZ00000000.1); M40553 (NZ_QQKO00000000; url - https://www.ncbi.nlm.nih.gov/nuccore/NZ_QQKO00000000.1); M40463 (NZ_QQHX00000000; url - https://www.ncbi.nlm.nih.gov/nuccore/NZ_QQHX00000000.1); *Neisseria polysaccharea* strains C2013011231 (NZ_POXT00000000; url - https://www.ncbi.nlm.nih.gov/nuccore/NZ_POXT00000000.1); NS342 (NZ_AEPH00000000; url-https://www.ncbi.nlm.nih.gov/nuccore/NZ_AEPH00000000.1); *Neisseria dumasiana* strains 114 725 (NZ_MTAA00000000; url - https://www.ncbi.nlm.nih.gov/nuccore/NZ_MTAA00000000.1); 124 861 (NZ_MTAB00000000; url - https://www.ncbi.nlm.nih.gov/nuccore/NZ_MTAB00000000.1); 93 087 (NZ_MTAC00000000; url - https://www.ncbi.nlm.nih.gov/nuccore/NZ_MTAC00000000.1); *Neisseria zoodegmatis* strains NCTC12229 (NZ_UGRS00000000; url - https://www.ncbi.nlm.nih.gov/nuccore/NZ_UGRS00000000.1); NCTC12230 (NZ_LT906434; url -https://www.ncbi.nlm.nih.gov/nuccore/NZ_LT906434); DSM 21643 (NZ_MTBM00000000; url -https://www.ncbi.nlm.nih.gov/nuccore/NZ_MTBM00000000.1); *Neisseria canis* strains ATCC 14687 (NZ_MTBL00000000; url - https://www.ncbi.nlm.nih.gov/nuccore/NZ_MTBL00000000.1); NCTC10296 (NZ_LR134313; url - https://www.ncbi.nlm.nih.gov/nuccore/NZ_LR134313); *Neisseria wadsworthii* strains DSM 22245 (NZ_CP059565; url - https://www.ncbi.nlm.nih.gov/nuccore/NZ_CP059565); 9715 (NZ_AGAZ00000000; url -https://www.ncbi.nlm.nih.gov/nuccore/NZ_AGAZ00000000.1); *Neisseria animaloris* strains C2015003240 (NZ_POYC00000000; url - https://www.ncbi.nlm.nih.gov/nuccore/NZ_POYC00000000.1); DSM 21642 (NZ_MTBN00000000; url - https://www.ncbi.nlm.nih.gov/nuccore/NZ_MTBN00000000.1); C2012029644 (NZ_POXR00000000; url - https://www.ncbi.nlm.nih.gov/nuccore/NZ_POXR00000000.1); *Neisseria zalophi* strain ATCC BAA-2455 (NZ_CP031700; url - https://www.ncbi.nlm.nih.gov/nuccore/NZ_CP031700);

The authors confirm all supporting data, code and protocols have been provided within the article or through supplementary data files.

**Impact Statement**

Commensal *Neisseria* spp. have been shown to outcompete pathogens of the same family using several different mechanisms including the Type VI Secretion System (T6SS). Here are described two different T6SS types across *Neisseriaceae *; T6SS-A and T6SS-B as well as the T6SS-Bi subtype of T6SS-B. An array of different putative effectors have been identified that include LysM domain proteins, hydrolases, phospholipases, and nucleases that are thought to be anti-bacterial in nature. Based on the current genomic data, T6SSs are found only in the non-pathogenic *Neisseria* spp. Future investigations into the T6SS of commensal *Neisseria* spp. and their secreted effectors may provide an avenue for the development of novel therapeutic options in the treatment of multidrug resistant infections.

## Introduction

Up to ten distinct bacterial secretion pathways have been discovered to date. Of the Sec-independent, one step systems that facilitate transport across both membranes of Gram-negative species, the Type VI Secretion System (T6SS) is one of the most recent [1–6].

While the T6SS was first defined functionally in the Gram-negative *Vibrio cholerae* by Pukatzki *et al*. [7], type VI genes had been identified previous to this in *Salmonella enterica* [8], *Rhizobium leguminosarum* [9], *Edwardsiella tarda* [10] and *Francisella tularensis* [11]. Additionally, as far back as 1996, the haemolysin co-regulated protein (Hcp) was identified to be secreted by a system that was at the time unidentified [12].

T6SS are large multiprotein complexes that bridge the cell envelope of Gram-negative bacteria [13]. These secretion systems are composed of 13 core proteins [14, 15] identified according to the standard T6SS nomenclature [16] as TssA, TssB, TssC, TssD (Hcp), TssE, TssF, TssG, TssH (ClpV), TssI (VgrG), TssJ, TssK, TssL, and TssM. While T6SS core genes are highly conserved [14, 17], effector loci have also been identified with genes that are ordered identically across different bacterial species [18].

In a functioning T6SS, TssABCDEFGHIJKLM form a structure that is similar to an inverted bacteriophage tail [15]. These 13 proteins are divided into three groups based on the T6SS subunit they co-assemble: the baseplate (TssEFGK); membrane complex (TssJLM); and the injection apparatus containing the needle sheath (TssBC).

While TssA is documented in some literature as being a component of the T6SS baseplate [15, 19–21], the role of this protein is slightly more complex. Zoued *et al*., (2016) showed that TssA interacts with TssJM in the early stage of T6SS biogenesis and is involved in the recruitment and positioning of the secretion system baseplate. TssA also initiates the coordinated assembly and extension of the tail tube/sheath [22, 23] and may also play a role in maintaining sheath stability while in an extended state [24].

Contraction of the TssBC sheath drives a structure consisting of stacked tubular TssD (Hcp) proteins topped with TssI from the secreting cell into target cells. TssI is a valine-glycine repeat (VgrG) protein ‘spike’ [25]; VgrG facilitate toxin effector secretion [26] and, following their delivery into target cells, the contracted TssBC sheaths are disassembled through recruitment of the AAA +ATPase, TssH (ClpV) [27, 28].

VgrG are both structural as well as secreted components of the T6SS [29], however, these genes are often not as well conserved as the ones encoding the other core secretion system components [30]. Within different bacterial species, *vgrG* can be present either individually or in multiple copies [31] and their gene products associate as either homotrimeric or heterotrimeric structures [32, 33] to form the puncturing device tip.

VgrG trimers are often capped by proline-alanine-alanine-arginine (PAAR) repeat motif containing proteins. While binding of VgrG with PAAR motif proteins gives the T6SS puncturing device a sharpened point [34], PAAR are also involved in the recruitment of certain effector proteins [35, 36].

It has been speculated that the T6SS evolved initially for the purpose of competitive fitness [20] with species adapting their T6SS over time to mediate interactions with other cell types [37]. It is thought that T6SS mediated killing of neighbouring cells could lead to the acquisition of new EI pairs through horizontal gene transfer (HGT) [38]. The acquisition of new EI pairs would allow bacterial populations to diversify, enhance niche survival [39, 40], and increase fitness in comparison to a parent strain [38, 40].

T6SS are versatile systems that have been shown to secrete a range of effector proteins including lipases, amidases, and nucleases [18, 41], as well as other pore forming toxins [37]. While T6SS are known to translocate toxins into fungal [42] as well as prokaryotic and eukaryotic cells [43], these systems may have other functions that include biofilm formation, host cell adhesion, and nutrient acquisition [44–47].

In relation to T6SS-mediated competition, many of the effectors already characterised have been shown to exert intraspecies [25] as well as interspecies antibacterial effects on other bacteria [20, 48]. Antibacterial T6SS have been identified in a range of Gram-negative species including *Serratia marcescens* [49], *Acinetobacter baumannii* [50]*, Klebsiella pneumoniae* [51, 52], *V. cholerae* [53], and *Neisseria cinerea* [54]. Overall, the T6SS may be responsible for shaping the composition of microbial populations [20, 41, 55, 56].

Commensal bacteria are known to be able to interfere with the capacity for host colonisation by pathogenic species. In regard to the commensal *Neisseria* spp., these have been shown to be able to out compete the pathogens using several mechanisms including the T6SS [54, 57, 58]. While interspecies competition within the gut and intestinal niches are well documented [59, 60], similar processes are thought to occur at other sites with a high turnover of colonising species including the pharyngeal mucosa and within the nasopharynx [61, 62].

Individual T6SS genes have previously been identified in *Neisseria mucosa, Neisseria sicca*, *Neisseria subflava* [63], *N. cinerea* [54], and two complete T6SS system types were described by Calder *et al*. [64] in isolates of *N. subflava *. While commensal *Neisseria* species are normally considered harmless, a study carried out by Li *et al*. [65] implicated *N. subflava* as an opportunistic pathogen being the cause of epithelial cell-cell barrier disruption and induction of inflammation in subjects with bronchiectasis. A small number of other disease cases have been reported; the case report and review carried out by Baraldes *et al*. [66] summarises details for eight well-documented cases of meningitis caused by *N. subflava *.

A full analysis of the presence and diversity of the *Neisseria* spp. T6SS has been undertaken to investigate the repertoire of these Gram-negative diplococci possessing these secretion systems. The core genes encoding the T6SS mechanism (*tssA*, *tssB*, *tssC*, *tssD*, *tssE*, *tssF*, *tssG*, *tssH*, *tssI*, *tssJ*, *tssK*, *tssL*, *tssM*) have been sought in the *Neisseria* spp. genomic data and their sequence homology, organization, and synteny assessed. Genes encoding PAAR and putative effector-immunity protein pairs for *N. subflava* strain KU1003-01 have also been assessed here and analysed in light of their presence within other *Neisseria* spp. Generation of a portfolio of T6SS effectors across *Neisseria* spp. will only be possible following the availability of more complete whole genome sequence data; this remains a subject for future investigations.

## Methods

### *In silico* identification of T6SS loci

T6SSs were identified in the Illumina MiSeq generated data that was assembled and annotated as described previously [64]. To gain greater clarity as to the chromosomal organization of the T6SS core genes and *vgrG* copy numbers within *N. subflava* strain KU1003-01, this genome was re-sequenced via MicrobesNG (Birmingham, UK) enhanced genome sequencing combining MiSeq and MinION short and long read technologies. The complete, circular chromosomal sequence (SUB4831743) was annotated using Prokka 1.14.3 [67] and visualised using DNA Plotter [68].

For comparative analysis the complete genome sequences of *N. subflava* strain KU1003-01, *N. subflava* strain M18660, and *N. subflava* strain ATCC 49275 were re-annotated using RASTtk [69, 70]. T6SS gene clusters were sought in these genome sequences using the T6SS-HMMER gene-finding tool on the SecReT6 database [71]. The 13 core T6SS genes identified by T6SS-HMMER in each of the three genome sequences were then located within the RAST annotations and the function of their predicted proteins was further investigated using BLASTp [72], and WU-blast 2.0 search against SecReT6 v2.0 database [71]. The 13 core T6SS genes from each of the three genome sequences were aligned using clustal O (1.2.4) [73].

To identify other *Neisseria* spp. possessing T6SS genes, T6SS genes (*tssA*, *tssB*, *tssC*, *tssD*, *tssE*, *tssF*, *tssG*, *tssH*, *tssJ*, *tssK*, *tssL*, *tssM*) from *N. subflava* strain KU1003-01 and from the reference genome *N. subflava* strain ATCC 49275 were used as query sequences in BLASTn searches [72]. For the *Neisseria* spp. on NCBI and PubMLST where blast hits were identified, the genome sequence data was downloaded and re-annotated using RASTtk for further investigation.

### Identification of genetic islands

Island Viewer 4 [74] was used to identify the presence of genomic (GI) or pathogenicity islands (PAI) in *N. subflava* strain KU1003-01, *N. subflava* strain M18660, and *N. subflava* strain ATCC 49275. Identified islands were assessed using DNA plotter’s GC skew function [68].

### T6SS diversity

To assess T6SS diversity, amino acid sequences for T6SS core components were evaluated in a Neighbour Joining tree using Unipro UGENE v38.1 [75]. Due to the coding sequences for TssH (ClpV) and TssI (VgrG) being interrupted by contig breaks for some genome sequence datasets, these were not included, therefore 11 of the 13 core T6SS amino acid sequences were concatenated using the combine fasta tool, https://www.bioinformatics.org/sms2/combine_fasta.html [76].

### Identification of T6SS-associated VgrG

Within the MinION sequence data for *N. subflava* strain KU1003-01, a total of nine *vgrG* CDS were auto-annotated by RASTtk. One truncated *vgrG* was identified within the original Illumina Miseq data that could not be identified in the MinION data. The amino acid sequences for a total of ten VgrG were aligned using clustal O (1.2.4) and percent identity matrices produced using Clustal Omega 2.0 [73].

The Simple Modular Architecture Research Tool (SMART) webserver, version 9.0 [77] and Phyre^2^ [78] was used to identify the presence of typical domains associated with VgrG. The domains were aligned using clustal O (1.2.4) and their similarities calculated using the Clustal Omega 2.0 % identity matrix tool. Based on this analysis, the diverse VgrG C-terminal regions following DUF2345 in *N. subflava* strain KU1003-01 were used as queries in tBLASTn to identify similar sequences in other *Neisseria* spp.

### PAAR

A gene encoding a DUF4150 containing protein was identified in the reference genome sequence of *N. subflava* strain ATCC 49275 (Genbank id: QCL71082.1). Proteins with DUF4150 are associated with PAAR in other species [79] and so the QCL71082.1 sequence was chosen to search for homologues across other *Neisseria* spp. genomes.

### Identification of T6SS chaperone proteins

T6SS chaperone proteins can be encoded in gene clusters along with *vgrG* and effectors [80], these proteins have been identified with domains DUF1795 [81], DUF2169 [80], and DUF4123 [82]. CDS FAH66_RS06000 in *N. subflava* strain ATCC 49275 is homologous to DUF2169 and is adjacent to a *vgrG*. DV114_RS04590 in *N. subflava* M18660 is homologous to DUF4123 and is also adjacent to *vgrG* within a polymorphic toxin locus. Predicted protein sequences from these CDSs were used as queries in tBLASTn searches to identify putative T6SS chaperones in *Neisseria* spp. No DUF1795 homologues could be identified in T6SS clusters for *N. subflava* strain KU1003-01 or either of the two *N. subflava* reference genome sequences.

In addition to the homology searching described above, the predicted products of CDSs both 5′ and 3′ of *vgrG* copies for *N. subflava* strain KU1003-01 and *N. subflava* strain M18660 were investigated using BLASTp [72] and the SMART webserver, version 9.0 [77] to identify chaperone protein associated domains.

### Effector proteins

To further investigate CDSs adjacent to *vgrG* for putative T6SS effectors, CDSs predicted to be co-expressed along with copies of *vgrG* based on operon predictions by FgenesB [83] and Operon Mapper [84], as well as the presence or absence of promoter sequences, predicted by Bprom [83] were investigated.

The products of CDS predicted to be co-expressed along with *vgrG* were subject to SMART analysis [77] to identify the presence of enzymatic domains. WU-blast 2.0 searches against the SecReT6 database [71] were carried out to identify homology to any known T6SS effectors and Bastion6 [85] used to compare features of these sequences to any previously identified T6SS effectors.

### Immunity proteins

WU-blast 2.0 [71] searches against the SecReT6 database were carried out to identify T6SS immunity protein. Unlike T6SS effectors, immunity proteins do not usually have N-terminal signal peptide sequences [86] and SignalP-5.0 [87] was also used to predict the presence or absence of signal peptide sequences for the products of CDS both 5′ and 3′ of *vgrG*.

## Results and discussion

Prokaryotic genomes can contain numerous repeated elements [88] that are either identical to one another or highly similar to other sequences at other genomic locations. Examples of repeated elements include rhs family genes, mobile genetic elements as well as genes encoding toxin-antitoxin systems [88–90]. Repetitive sequences are known to cause issues during whole genome assembly [91] and *de novo* sequence assemblers can struggle to distinguish small differences between them [92]. In some instances, these sequences can mean that the task of complete genome assembly is not possible [93].

Within the original Illumina MiSeq sequencing data for *N. subflava* strain KU1003-01 [64], *vgrG* and genes encoding mobile element proteins fragmented the sequence assembly. Out of 58 assembled contigs, 11 contigs terminated at mobile element genes and 21 at *vgrG*. Numerous contigs terminating with *vgrG* are also present within the draft genome sequences of other *N. subflava* biovar *perflava* and *N. flavescens* (data not shown), thus highlighting the difficulties caused by multiple copies of homologous, repetitive sequences during whole genome sequence assembly [94].

It was for this reason that enhanced genome sequencing was chosen for *N. subflava* strain KU1003-01 with a hybrid strategy for genome assembly that involved combining Illumina short reads with the longer MinION reads. Accurate assessments of *vgrG* copies, and associated sequences such as effectors and immunity (EI) genes in *N. subflava* strain KU1003-01, was only possible following analysis of the complete hybrid strategy-generated genome sequence.

**Fig. 1.**
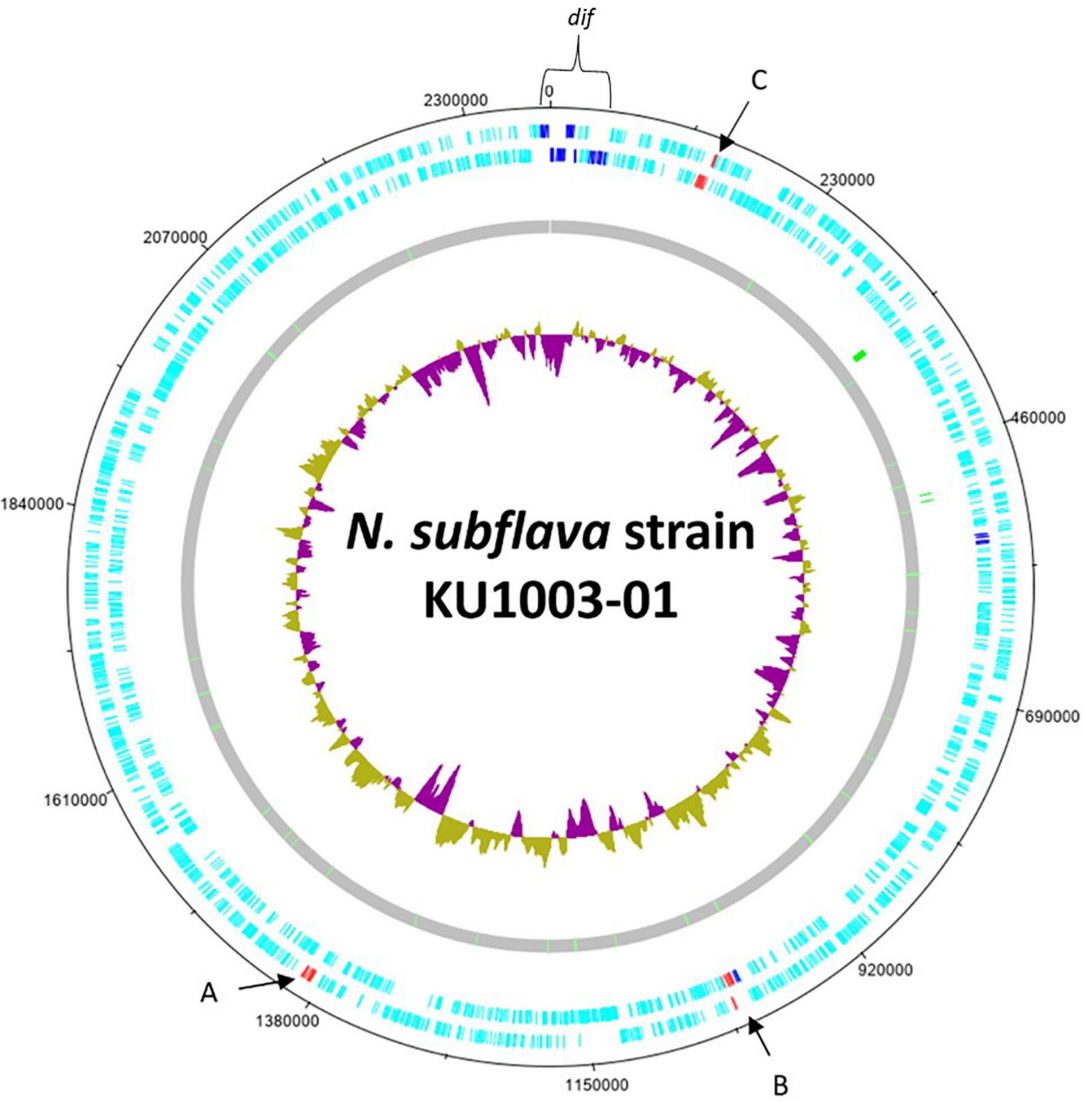
A circular representation of the complete *N. subflava* strain KU1003-01 genome created using DNAPlotter [68]. Annotated CDSs are shown in the outer two rings (light blue, dark blue, red) and tRNA loci within the inner grey circle (green). The inner circle (purple and gold plots) represents chromosomal GC content, purple indicates regions that are below the chromosomal average and gold are those above the chromosomal average. The T6SS-A core gene clusters (red) are in two locations on the chromosome (**a and b**) with a duplication of one of the clusters (**c**). The *vgrG* EI clusters (dark blue) are associated with one of the core gene clusters (**b**) and elsewhere on the genome. The genomic region marked ‘dif’ represents the gene clusters where *dif* sequences are present.

The complete genome sequence for *N. subflava* strain KU1003-01 reveals T6SS core gene clusters present at two locations on the chromosome (Fig. 1). A duplication of one of the core gene clusters containing copies of *tssH*, *tssD*, *tagL*, *tssL*, *tssK*, *tssB*, and *tssC* is present at positions 129 554 to 138 892. Alignment of the duplicate region to the cluster present between positions 1 018 014 to 1 030 292 identified these regions as having 98 % nucleotide identity to one another according to a Percent Identity Matrix - created by Clustal 2.1 suggesting this 9.3 kb region has arisen through duplication.

A duplicate core T6SS gene cluster is also present in the reference genome sequence of *N. subflava* strain M18660. It is anticipated that identical or near identical duplications such as the one seen in *N. subflava* strain KU1003-01 would not be readily identified in other draft genome sequence data. Indeed, this duplication was not evident in the draft data of *N. subflava* strain KU1003-01 [64]. Analysis of the number of T6SS core gene clusters across complete commensal *Neisseria* spp. genomes with the same T6SS type including *N. mucosa* strain FDARGOS_260, *N. mucosa* strain ATCC 29256, *N. elongata* strain M15910, and *N. sicca* strain DSM 17713 indicates a duplicate core T6SS-A gene cluster is likely to only be present in *N. subflava* biovar *perflava* and *N. flavescens *.

The core T6SS genes of *N. subflava* strain KU1003-01 share between 97–100 % identity to those in the reference genome *N. subflava* strain M18660 but only share between 52–57 % identity to those of *N. subflava* strain ATC 49275. The core genes of *N. subflava* strain RH3002v2g share between 93–97 % identity with the core genes of *N. subflava* strain ATC 49275 and only 50–56 % identity to those of *N. subflava* strain M18660 (Table 1).

**Table 1.** T6SS-A core genes of *N. subflava* strain KU1003-01 compared to T6SS-A of *N. subflava* strain M18660 and T6SS-B of *N. subflava* strains ATCC 49275, KU1003-02, and RH3002-v2g

Gene	Locus tag: *N. subflava* strain KU1003-01	T6SS core component	Gene product			
			% similarity to *N. subflava* strain KU1003-01			
			* N. subflava* strain M18660	* N. subflava* strain ATCC49275	* N. subflava* strain KU1003-02	* N. subflava* strain RH3002v2g
*tssA*	23 676E_KU100301_01345	Secretion system biogenesis.	100	23.51	23.18	22.85
*tssB*	23 676E_KU100301_00982	Contractile sheath - small subunit	100	29.45	30.49	30.49
*tssC*	23 676E_KU100301_00983	Contractile sheath - large subunit	100	40.49	40.69	40.69
*tssD*	23 676E_KU100301_00978	Puncturing device inner tube	100	24.46	24.46	24.46
*tssE*	23 676E_KU100301_01349	Baseplate component	100	23.91	22.39	22.39
*tssF*	23 676E_KU100301_01346	Baseplate component	100	26.25	26.42	25.85
*tssG*	23 676E_KU100301_01347	Baseplate component	100	21.04	21.34	21.04
*tssH*	23 676E_KU100301_00977	Cytoplasmic T6SS protein recycling ATPase	99	45.51	45.85	45.85
*tssJ*	23 676E_KU100301_01348	Outer membrane lipoprotein	100	22.16	22.16	21.56
*tssK*	23 676E_KU100301_00981	Baseplate component	99	23.06	23.11	23.11
*tssL*	23 676E_KU100301_00980	Inner membrane / TM complex	100	19.44	19.91	19.91
*tssM*	23 676E_KU100301_01344	Inner membrane / TM complex	100	20	19.8	19.8
ompA*	23 676E_KU100301_00979	Possible T6SS translocation pore	100	NP	NP	NP

*TssL in KU1003-01 does not have PGB domains, unlike the ‘evolved TssL’ types [13], these domains are encoded separately by *tagL*.

**Fig. 2.**
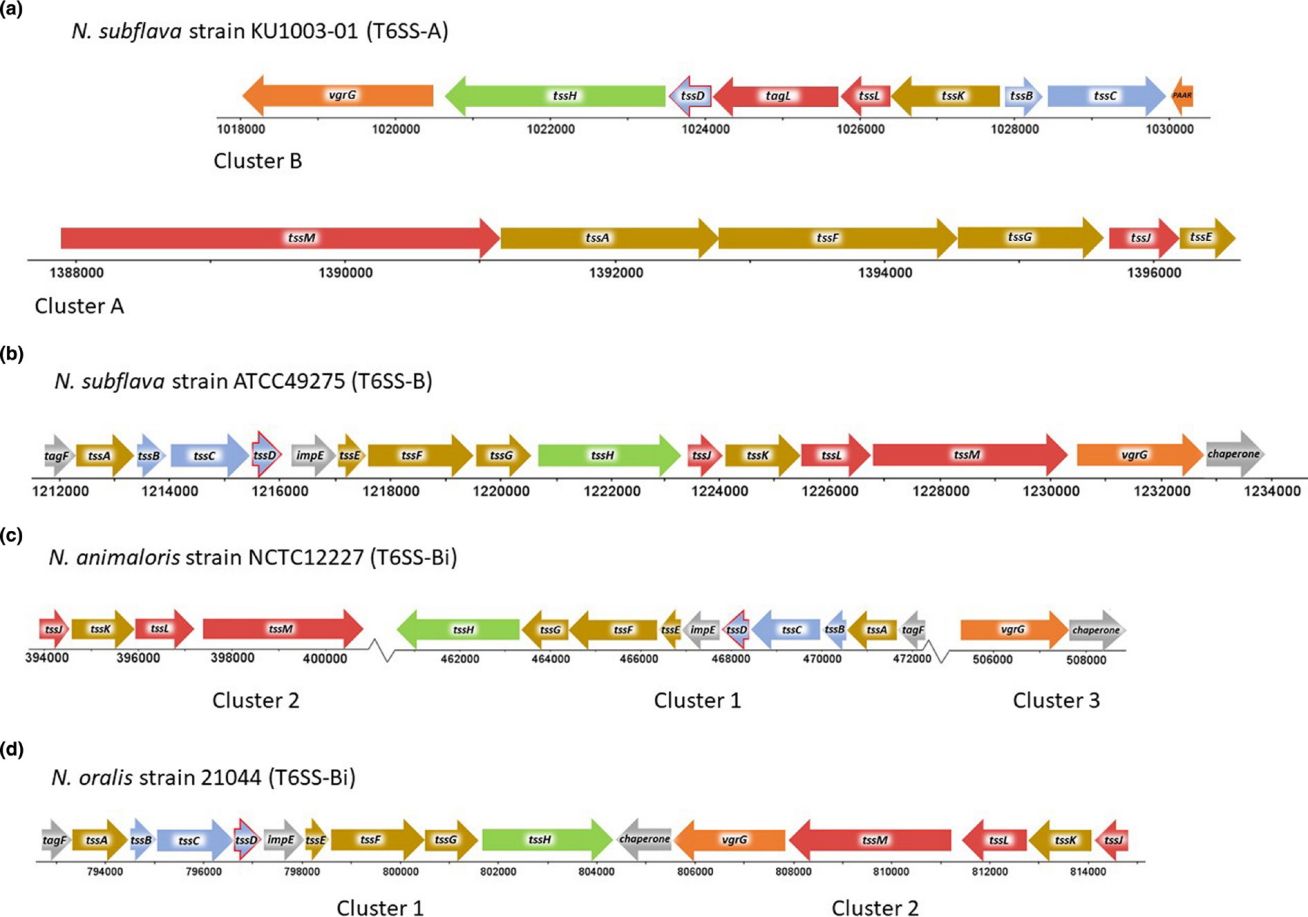
The *Neisseria* spp. Type VI Secretion System core gene clusters. (**a**) Organisation of the T6SS-A core genes in *N. subflava* strain KU1003-01. (**b**) The T6SS-B in the reference genome sequence of *N. subflava* strain ATCC49276. (**c**) The T6SS-Bi subtype in *N. animaloris* strain NCTC12227 and (**d**) *N. oralis* strain 21 044. *N. subflava* strain KU1003-02 and RH3002-v2g have identically organised T6SS to that shown in panel b.

Two main T6SS types, T6SS-A and T6SS-B, have been identified in *Neisseria* spp., as evidenced by differences in core gene sequence homology and organization (Fig. 2a, b). *N. subflava* strains KU1003-01 and M18660 have T6SS-A. *N. subflava* strains KU1003-02, RH3002v2g, and ATCC 49275 have T6SS-B.

### Analysis of genome sequence data on the NCBI and PubMLST databases

Of the 259 T6SS possessing neisserial genome sequences identified on both NCBI and PubMLST, as of August 2021, only ten are complete, closed, circular genome sequences. Complete T6SSs were identified in 59 genome sequences on NCBI and 200 on PubMLST (Table S1). Of the 259 genome sequences, 27 have T6SS-A only, with similarity to the systems seen in *N. subflava* strains KU1003-01 and M18660, and 197 genomes have T6SS-B only. A further 27 isolates across both NCBI and PubMLST have both T6SS types within their genome sequences. The remaining eight genome sequences have a subtype of T6SS-B, identified here as T6SS-Bi.

TSS6-B appears to be the predominant type in *Neisseria* spp.; 13 species are identified as only having T6SS-B. There are six *Neisseria* spp. that have only T6SS-A and two with the subtype T6SS-Bi. Of the *Neisseria* spp. identified with both T6SS-A and T6SS-B within a single genome sequence, these include: *Neisseria canis; Neisseria dumasiana; Neisseria macacae; Neisseria sicca;* and *Neisseria zoodegmatis *. The identification of two different T6SS types within a single genome is not unique, as previously *Escherichia coli* [95], *Azoarcus olearius* [96], and *Vibrio parahaemolyticus* [97] have been identified with more than one T6SS type.

While T6SS are a common feature of commensal *Neisseria* spp., following BLASTn searches for T6SS core genes in around 10 000 *N *. *gonorrhoeae* genome sequences and 29 000 *N *. *meningitidis* genome sequences across both PubMLST and NCBI, these secretion systems could not be identified in the pathogens. In the absence of T6SS, it is likely the pathogens rely on other mechanisms for competition within their niche.

In regard to *N. meningitidis *, all isolates are thought to carry at least one contact dependant inhibition (CDI) system with some strains having multiple loci consisting of a large number of CDI-associated toxin/immunity genes [98]. CDI systems are two-partner secretion mechanisms (TPS), a branch of type V secretion systems [99] that are known to regulate growth of neighbouring cells through cell to cell contact [100].

Unlike *N. meningitidis *, *N. gonorrhoeae* are not thought to have functioning CDI systems [101]. There are few studies on the ability of *N. gonorrhoeae* to outcompete other species although it has been suggested that *N. gonorrhoeae* may facilitate its survival at the expense of its neighbours through modulation of polymorphonuclear leukocytes (PMN) biology [102]. At specific stages of disease, modulation of the PMN oxidative burst can aid gonococcal survival [102, 103].

### Neisserial T6SS and their similarities to SecReT6 types

SecReT6 [104, 105] has classified T6SSs from various Gram-negative species into three types: type i; type ii; and type iii. The Type i T6SS are further subdivided into six subtypes: i1; i2; i3; i4a; i4b; and i5. According to T6SS classification predicted through the SecReT6 webserver, the neisserial T6SS-A is similar to the SecReT6 T6SS subtype i2. Since initial identification of T6SS-A and T6SS-B [64], a subtype of T6SS-B (T6SS-Bi) has been discovered within genome sequences of *Neisseria animaloris* and *Neisseria oralis *. The T6SS-B and T6SS-Bi subtypes are similar to members of the SecReT6 T6SS i3.

**Fig. 3.**
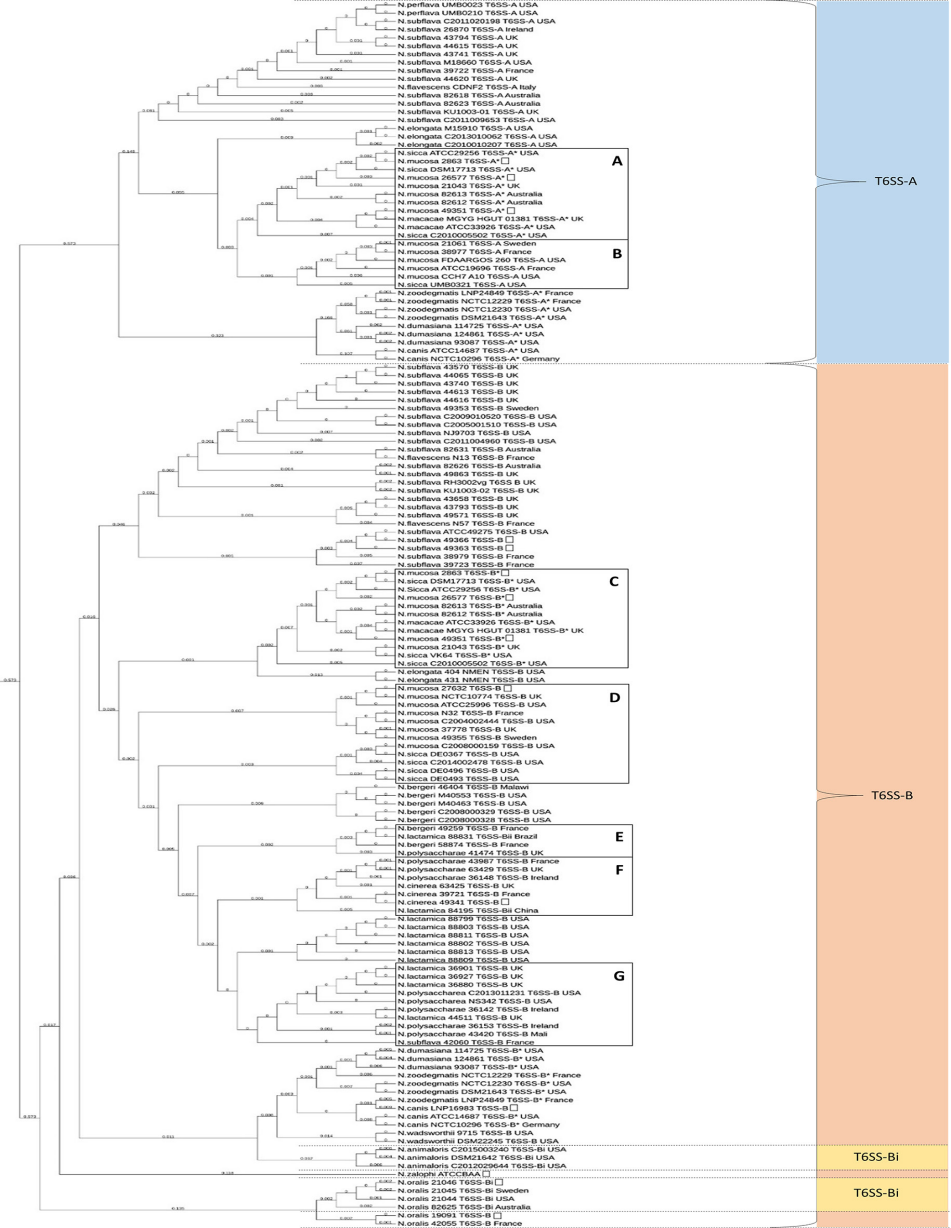
Neighbour Joining tree constructed following alignment of 11 concatenated *Neisseria* spp. T6SS core gene products using Ugene [75]. The tree image was formatted using the online Interactive Tree of Life (iTOL) tool [158]. (**a**) Eleven T6SS-A genome sequences believed to be *N. sicca *. (**b**) Six T6SS-A genome sequences believed to be *N. mucosa *. (**c**) Twelve T6SS-B genome sequences believed to be *N. sicca *. (**d**) Twelve T6SS-B genome sequences believed to be *N. mucosa *. (**e**) Evidence of sharing of T6SS-B core components between *N. bergeri, N. lactamica,* and *N. polysaccharae*. (**f**) Evidence of sharing of T6SS-B core components between *N. cinerea, N. lactamica, and N. polysaccharae*. (**g**) Lone *N. subflava* (strain 42060) with T6SS-B gene products 99 % similar to those in *N. polysacchareae* and *N. lactamica *. * Genome sequences with both T6SS types A and B. 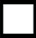 Geographical origin not available.

The core gene products of T6SS-Bi of *N. animaloris* are on average 85 % sequence identical to those of *N. subflava* strain ATCC 49275 and around 92 % sequence identical to those of *N. canis *, *N. dumasiana,* and *N. zoodegmatis *. For *N. oralis,* these are 76 and 78 % sequence identical, respectively. While these T6SSs have sequence homology to T6SS-B, their core genes are organised differently (Fig. 2c, d). The clustering of the T6SS-Bi in relation to the other neisserial T6SSs is shown on a Neighbour Joining tree (Fig. 3).

### Characteristics of T6SS-A

The genetic organisation of T6SS-A is conserved within *Neisseria* spp. where T6SS-A is the sole type present (Table S1; Fig. 2a). T6SS-A have their core genes present across two loci with at least one *vgrG* and a gene encoding PAAR located with one of the core gene clusters (Fig. 2a). T6SS-A also have ‘orphan’ copies of *vgrG* scattered on their chromosomes, separate from the core gene clusters. While *N. subflava* strain KU1003-01 has a total of ten T6SS-A type *vgrG* copies (Fig. 1), investigations from other complete *Neisseria* spp. genomes highlight a varying number of *vgrG* copies. For example, *N. subflava* strain M18660 has six *vgrG* copies, *N. mucosa* strain FDARGOS_260 has 11, *N. mucosa* strain ATCC19696 has nine, *N. elongata* strain 15 910 has ten, *N. macacae* strain ATCC33926 has three, *N. canis* strain NCTC10296 has four, and *N. zoodegmatis* strain NCTC12230 has eight copies of *vgrG*.

Within *N. subflava* strains KU1003-01 and M18660 and strains of *N. elongata *, a single PAAR CDS is located at one of the core clusters. For *N. mucosa *, two putative PAAR are present, with one present at each core gene cluster. For the *N. mucosa* strains highlighted in Box B of the Neighbour Joining tree (Fig. 3) one of these PAAR CDSs is ‘disrupted’ by a 107 bp Correia Repeat-Enclosed Element (CREE) [106, 107]. CREE are known to disrupt coding sequences [108] and to include promoters [109, 110]. This CREE disrupted PAAR copy was not auto-annotated by either Prokka 1.14.3 [67] or RASTtk [69, 70], likely due to an ATg to ATc start codon mutation caused by one of the CREE inverted repeats. Although the CREE does have upstream sequences similar to the promoters identified in *N. gonorrhoeae* by [109], it is not clear from this study if this copy of PAAR would be functional.

All T6SS-A have a peptidoglycan binding (PGB) domain protein, OmpA (*tagL*) encoded between *tssL* and *tssH*. While inner membrane proteins (TssL) have been identified both with and without PGB domains [13] for different T6SS types, the T6SS-A type TssL do not have PGB domains. In neisserial T6SS-A, the PGB domain is encoded separately as the accessory protein *tagL* [111].

The exact role of TagL within the commensal *Neisseria* spp. is unclear. While OmpA domain proteins can be found within T6SS gene clusters for all virulent strains of *E. coli, Burkholderia, Yersinia, Pseudomonas,* and *Ralstonia* [86, 112], these accessory proteins are thought to be involved in localising the secretion system to specific cellular sites [111] and stabilising its structure [13], or forming T6SS membrane translocation pores [86].

### Characteristics of T6SS-B

The genetic organisation of the T6SS-B is conserved across the *Neisseria* spp. investigated (Table S1; Fig. 2b). *N. subflava* strains KU1003-02, RH3002v2g [64], and ATCC 49275 all have T6SS-B. T6SS-B are characterised by the presence of a single T6SS gene cluster adjacent to a gene encoding regulatory protein TagF with DUF2094 [113]. The T6SS-B in *N. subflava* strains KU1003-02 and RH3002v2g [64] are arranged identically to the T6SS identified on a plasmid of *N. cinerea* by Custodio *et al*. [54].

All *Neisseria* spp. with T6SS-B have a single *vgrG* followed by a gene encoding a putative chaperone protein with a DUF2169-like domain [80], these are encoded downstream of *tssM* at one end of the core cluster. In *N. subflava* strain ATCC 49275, the organisation of genes downstream from *vgrG* and the putative chaperone bear similarities to the polymorphic toxin loci described by [114].

For *N. subflava* strains KU1003-02, RH3002v2g, and ATCC 49275, as well as the other *Neisseria* spp. investigated here, T6SS-B clusters are flanked at either end by *rhs*. In *N. subflava *, the Rhs encoded downstream of *vgrG* have N-terminal DUF4150 - PAAR-like domains, however, these domains are absent for the Rhs encoded upstream of *tssA*. While these findings are similar to those of Custodio *et al*., [54] for *N. cinerea *, for *N. subflava* strains RH3002v2g and ATCC 49275, additional, ‘orphan’ *rhs* with PAAR domains are present elsewhere on their chromosomes and away from the core T6SS cluster.

Unlike the T6SS-A, T6SS-B core clusters do not feature *tagL*. Instead, these systems have ‘evolved’ TssL [13, 115] with C-terminal OmpA PGB motifs. The T6SS-B also feature an accessory protein ImpE, encoded between *tssE* and *tssD*. In some species, ImpE is involved in temperature dependent secretion as well as virulence [8, 86].

### Characteristics of T6SS-Bi

T6SS-B subtypes could only be identified in *N. animaloris* (Table S1; Fig. 2c) and *N. oralis* (Table S1; Fig. 2d). For *N. animaloris *, the core T6SS-Bi genes are separated into three clusters. Cluster one consists of *tagF*, *tssA*, *tssB*, *tssC*, *tssD*, *impE*, *tssE*, *tssF*, *tssG,* and *tssH* (*clpV*). Cluster two is *tssJ*, *tssK*, *tssL*, and *tssM*. Cluster three consists of *vgrG* and the putative chaperone gene. For *N. oralis *, the core genes are separated into two clusters. Cluster one consists of *tagF*, *tssA*, *tssB*, *tssC*, *tssD*, *impE*, *tssE*, *tssF*, *tssG,* and *tssH* (*clpV*). Cluster two is *tssJ*, *tssK*, *tssL*, *tssM*, *vgrG,* and the chaperone gene.

T6SS-Bi clusters one and two are encoded on opposite strands for both *N. oralis* and *N. animaloris *. Within the genome sequences of *N. animaloris* the two clusters are separated by an approximately 60 kb region, the gene pair (cluster three), consisting of *vgrG* and chaperone is located over 30 kb away from cluster one. For *N. oralis,* the region separating clusters one and two is much shorter at only 78 bases. For both species, Rhs with N-terminal DUF4150 PAAR and toxin domains are not encoded downstream of *vgrG* although these are present within different regions of these chromosomes.

### Characteristics of T6SS-A and T6SS-B within *Neisseria* spp. genomes where both types are present

For the *Neisseria* spp. possessing both T6SS types (T6SS-A/B), including *N. canis, N. dumasiana, N. macacae, N. sicca*, and *N. zoodegmatis* (Table S1), the organisation of T6SS-B core genes are identical to those within the T6SS-B only isolates. For these species however, differences exist regarding their T6SS-A.

With the exception of *N. canis *, all T6SS-A/B *Neisseria* spp. have a full repertoire of 13 T6SS core genes in their T6SS-A. Within the complete genome sequence for *N. canis* strain NCTC 10296*, tssA* and *tssM* are not present and *tssF* is preceded by a putative transposase. While *N. canis* has VgrG encoded at one of the core clusters, for the other T6SS-A/B species, *vgrG* are not present at either core cluster. All of the T6SS-A/B species do however have ‘orphan’ T6SS-A type *vgrG* scattered throughout their chromosomes.

With the exception of *N. canis *, all T6SS-A/B *Neisseria* spp. have PAAR encoded at a different T6SS core cluster than the T6SS-A only species. Within the complete genome sequence for *N. canis* strain NCTC 10296, PAAR is encoded within an entirely different region of the genome to the core T6SS clusters and immediately upstream of a nucleoporin-like protein CDS. While *N. macacae* and *N. sicca* have a single T6SS-A type PAAR encoded at one of their core clusters, the draft genome sequences for *N. dumasiana* strain 93 087 and *N. zoodegmatis* strain NCTC12229 have a second ‘orphan’ PAAR with lower similarity to the one encoded at the core cluster. In *N. dumasiana* strain 93 087, the ‘orphan’ PAAR is followed by a putative T6SS amidase effector (Tae4) and immunity (Tai4) gene pair.

Within the genome sequences of the T6SS-A/B *N. sicca* and *N. macacae* investigated, both T6SS-A core gene clusters are flanked on either side by CREE with conserved CR inverted repeat sequences at either end [106, 107]. CREE are not present at the same positions within the *Neisseria* spp. that only have T6SS-A. Within the reference genome for *N. sicca* strain DSM 17713, the CREE flanking core cluster A are both 157 bp in length and those flanking core cluster B are 157 and 161 bp in length. All four CREE contain the ‘TAAGGTGCTGAAG’ IHF binding sequence described by Snyder *et al*. [107]. Overall, the CREE share between 97–100 % sequence identity across their full lengths. The inverted repeats at either end of these sequences differ by either one or two bases in comparison to the ‘TATAGTGGTTT’ described by Liu *et al*. [106] and consist of either ‘TATAGTGGaTT’ or ‘TATAtTGGaTT’.

While CREE are abundant in the pathogenic *Neisseria* spp., these are also the second most common repeat type found in the commensals [63]. CREE with similar lengths are present in both commensal and pathogenic species [106, 107] and in the pathogens, CREE are often identified near virulence or metabolic genes [106]. It is thought CREE may play a role in genome rearrangements as well as in gene regulation [107].

### Distribution of the T6SS across *Neisseria* species

The Neighbour Joining tree (Fig. 3) shows distinct patterns of clustering for the different neisserial T6SS types. Three main groups can be seen for T6SS-A. The first consists of *N. subflava* biovar *perflava* and *N. flavescens *, the second consists of *N. elongata, N. mucosa*, and *N. sicca,* and the third *N. zoodegmatis *, *N. dumasiana *, and *N. canis *, which are species commonly associated with non-human hosts.

*N. subflava* biovar *perflava* and *N. flavescens* are thought to have arisen from a common ancestor and clustering of these T6SS-A sequences as one group is consistent with these being variants of the same ancestral species [116].

Within the second group, additional genomic analyses were conducted to determine whether the annotated species designations were consistent with the homology data. The genome sequences for these *N. mucosa* and *N. sicca* T6SS-containing strains was analysed using the genome comparator tool at PubMLST for core genome MLST (cgMLST) and phylogenetic analysis [117] and aligned using the MAUVE contig mover (MCM) against reference genome sequences [118].

Based on the results from MCM, the draft genomic data from 11 stains (Fig. 3, Box A) aligned with greater collinearity across the entire genome to the reference genome sequence *N. sicca* strain DSM 17713. cgMLST analysis also clustered these isolates as a group along with the *N. sicca* reference genome. Therefore, six *N. mucosa* and two *N. macacae* are suggested by MCM alignments, CgMLST, and T6SS phylogeny to be *N. sicca* (Fig. 3, Box A). The genomes for the six strains highlighted by Fig. 3, Box B align closely with the reference genome for *N. mucosa* strain FDAARGOS 260. cgMLST analysis also clustered these *N. mucosa* isolates as a group along with *N. sicca* strain UMB0321.

The non-human *Neisseria* spp. including *N. zoodegmatis *, *N. dumasiana,* and *N. canis* are genetically distantly related to other *Neisseria* spp [116]. and share a similar environmental niche as inhabitants of the normal oropharyngeal flora of non-human hosts [119, 120]. TssB, TssC, TssD (Hcp), and TssE of *N. dumasiana* are homologous to those found in *N. zoodegmatis* and share between 97–100% similarity along their lengths. TssAFGJKLM however have lower similarity and are on average 86 % similar.

Certain components of the T6SS including TssB are highly conserved [121] and while the high level of similarity and phylogenetic clustering for the T6SS-A genes suggests common ancestry, there is no evidence of recent sharing of T6SS-A core genes across these species. The clustering of the *N. canis* T6SS-A is consistent with the similarity scores for these components. These are less similar to those of *N. zoodegmatis* and *N. dumasiana* and only share 72 % similarity along their lengths.

T6SS-A components are conserved across the commensal *Neisseria* spp. Homologues of TssB, TssC, TssK, and TssL with >45 % identity were identified in other genera, including the beta proteobacteria, *Chromobacteria* spp. Similar levels of homology were observed by [121] in their analysis of *Ralstonia solanacearum* and by Repizo *et al*., [122] when assessing the *Acinetobacter* genus.

The distribution of neisserial T6SS-B genes clusters into five main groups with the first group consisting of *N. subflava* and *N. flavescens *. The second and largest of the five groups consists of *N. sicca *, *N. macacae *, *N. elongata *, *N. mucosa *, *N. bergeri*, *N. lactamica *, *N. polysaccharea,* and *N. cinerea *. The third includes *N. dumasiana *, *N. zoodegmatis *, *N. canis *, and *N. wadsworthii *. Sharing a phylogenetic branch with the third group are *N. animaloris *, which are distinct in possessing the T6SS-Bi subtype where the gene organization differs (Fig. 2c). Interestingly, the T6SS-Bi type is also seen in *N. oralis *, which is on a distant phylogenetic branch in comparison to the other *Neisseria* spp. A lone representative of *Neisseria zalophi* also demonstrates the presence of the T6SS-B in more diverse *Neisseria* spp. *N. zalophi* was isolated from the oral cavity of a California Sea Lion (*Zalophus californianus*) [123]. With the exception of TssBCD of *N. zalophi* which are on average 97 % similar to those of *N. canis *, *N. dumasiana,* and *N. zoodegmatis *, the other T6SS components TssAEFGJKLM are on average 77 % similar to those within these non-human commensals.

Using MCM and cgMLST as for the T6SS-A possessing strains, 12 T6SS-B possessing strains believed to be incorrectly assigned species designations were investigated. Here six *N. mucosa* and two *N. macacae* are suggested to be *N. sicca* based on alignments from MCM, cgMLST results, and T6SS phylogeny (Fig. 3, Box C). Likewise, four *N. sicca* are believed to be *N. mucosa* (Fig. 3, Box D).

Interestingly, the application of this same analysis using the phylogenetic clustering data, MCM, and cgMLST determined that some T6SS-B groups contained mixtures of species (Fig. 3, Boxes E, F, and G). This highlights the sharing of T6SS-B core components through horizontal gene transfer events between *N. bergeri*, *N. lactamica *, and *N. polysaccharae* (Fig. 3, Box E). The latter two species are common commensal species of the human nasopharynx and are known to engage in frequent horizontal gene transfer events [124, 125] therefore this is perhaps not surprising.

The phylogenetic tree also highlights sharing between *N. polysaccharae*, *N. cinerea *, and *N. lactamica *, which is also in line with previous research focused on other features [63, 126, 127] (Fig. 3, Box F). Lastly, a cluster of similar T6SS-B possessing strains of *N. lactamica* and *N. polysaccharae* share a branch of the phylogenetic tree with a more distantly related *N. subflava* (Fig. 3, Box G). Upon investigation, this lone *N. subflava* strain 42 060 (LNP28165) fits amongst the members of the *N. subflava* species by cgMLST and MCM analysis. However, the T6SS-B gene products are 99 % similar as determined by Clustal 2.1 to those in *N. polysaccharea* and *N. lactamica *, yet only 90 % similar to *N. subflava* strain ATCC 49275.

The concatenated T6SS-B amino acid sequences from *N. dumasiana *, *N. zoodegmatis *, *N. canis *, and *N. wadsworthii* are between 98 and 100% similar to one another and suggests there may be horizontal gene transfer of T6SS-B genes between these species. These species’ T6SS-B proteins are around 91 % similar to those of the T6SS-Bi type proteins of *N. animaloris *.

The high level of homology for the T6SS components across different *Neisseria* spp., isolated from disparate geographical locations, and from different hosts living in diverse habitats suggests these T6SSs have been long established in the *Neisseria* spp. However, phylogenetic analysis does not indicate recent sharing of T6SS-A core components across the *Neisseria* spp. Mixed species phylogenetic branches suggest the situation is different for T6SS-B. For example, *N. subflava* strain 42 060 contains T6SS-B gene products more closely related to those in *N. polysaccharea* and *N. lactamica* than others of the same species (Fig. 3, Box G), indicative of horizontal gene transfer.

A T6SS-B has been identified on a plasmid of *N. cinerea* isolate 49 341 (CCUG 346T) by Custodio *et al*. [54], which may have provided a possible source of genetic material for horizontal gene transfer of the Type 6 Secretion genes. This *N. cinerea* isolate 49 341 (CCUG 346T) T6SS shares a branch on the phylogenetic tree with *N. lactamica* and *N. polysaccharae* (Fig. 3, Box F).

### Organization of *Neisseria* spp. T6SS loci

The separation of T6SS loci into multiple transcriptional units can give insight into how different T6SS gene arrangements and clusters have evolved across different species [122]. T6SS loci from other bacterial species have already been identified within co-regulated operons [128] and can be found encoded in separate transcriptional blocks in, for example, the *Acinetobacter* genus [122].

Given the organization of the neisserial T6SS-A into two gene clusters, with some including a duplication as in *N. subflava* strain KU1003-01, and the neisserial T6SS-B into one gene cluster, it is apparent that these different manifestations of the Type 6 Secretion Systems will also differ in the nature of their transcriptional units.

To investigate the potential transcriptional organization of the T6SS-A, FgenesB [83] and Operon Mapper analyses [84] were carried out on T6SS-A core clusters one and two of *N. subflava* strain KU1003-01. T6SS-A core cluster B is arranged into four putative operons according to FgenesB: operon one includes a hypothetical gene and PAAR; operon two consists of *tssC* and *tssB*; operon three includes *tssK*, *tssL*, *ompA*, *tssD*, and *tssH*; and operon four consists of *vgrG* and two hypothetical protein encoding CDSs. These predictions fit the gene orientations and spacing of the CDSs well (Fig. 2a). FgenesB predicted identical operons for the T6SS-A sequences in the reference genomes *N. mucosa* strain FDARGOS-260, *N. elongata* strain M15910, *N. sicca* strain DSM 17713, and *N. sicca* strain UMB0321.

Operon Mapper predicts just three operons for T6SS-A core cluster B, combining the FgenesB operon three and four. In support of these predictions, an Inverted Repeat (IR) of the neisserial DNA Uptake Sequence (DUS IR) is present in-between *clpV* and *vgrG*1. Although a quarter of all genes are thought to be either attenuated or terminated by IR sequences [129], according to ARNold [130], the IR following *clpV* is not a transcriptional terminator. RNA-seq work carried out by [131] in *N. gonorrhoeae* confirmed transcription can occur across DUS IRs and it is therefore possible, the IR at core cluster B acts as a transcriptional attenuator rather than a terminator.

T6SS-A cluster A of *N. subflava* strain KU1003-01 is encoded as a single operon consisting of *tssM*, *tssA*, *tssF*, *tssG*, *tssJ*, and *tssE* according to Operon Mapper; these predictions fit with the gene orientations and spacing of CDSs predicted by both RAST and Prodigal:2.6. In contrast, FgenesB analysis predicted T6SS-A cluster A to be encoded as two operons with the first containing *tssM*, *tssA*, and *tssF* and the second *tssG*, *tssJ,* and *tssE*. In support of the predictions of Operon Mapper*,* within the complete genomes of *N. mucosa* strain FDARGOS-260 as well as *N. elongata* strain M15910*,* cluster A are predicted to be encoded as a single operon according to both FgenesB and Operon Mapper.

According to FgenesB predictions, the T6SS-B of *N. subflava* ATCC 49275 is composed of four operons. Operon one consists of *tagF*, *tssA*, *tssB*, *tssC*, and *tssD*; this is followed by a single transcriptional unit consisting of *impE*. Operon two consists of *tssE*, *tssF*, *tssG*, and *tssH* and is followed by operon three consisting of *tssJ*, *tssK*, *tssL*, and *tssM*. FgenesB predicted *vgrG* as well as the gene encoding the putative chaperone protein with DUF2169 and *rhs* as operon four. In contrast to these predictions, Operon Mapper predicts the T6SS-B as being arranged as a single operon that includes *vgrG* and the downstream CDS encoding the chaperone protein. The FgenesB predictions are thought to accurately represent the operonic arrangement of T6SS-B on the basis of the arrangement of genes in T6SS-Bi.

In accord with the FgenesB predictions for the T6SS-B, the T6SS-Bi clusters for *N. animaloris* strain and *N. oralis* (Fig. 2c, d), are present as three and two separate clusters, respectively. In addition, while isolates of *N. oralis* can have either T6SS-B or T6SS-Bi (Table S1), the gene products for these two types are on average 99 % similar to one another. It is therefore most likely the T6SS-Bi subtypes within both species have arisen through rearrangement of a once single T6SS-B locus. Such rearrangements that retain function are more likely when the locus is composed of multiple operons than a single operon, which would require acquisition of new gene expression capacity after rearrangement.

The operon predictions and core gene clustering seen with T6SS-Bi are consistent with findings in other species. Repizo *et al*., [122] identified two different genetic arrangements of T6SS among *Acitenobacter* spp. that are thought to have arisen from a single T6SS loci being split into separate transcriptional blocks.

### VGRG C-terminal diversity

T6SS VgrG are multi-domain proteins that bear similarities to a polypeptide produced through fusion of two T4 phage gene products (gp), gp27 and gp5 [33], also known as gpD or gpV in bacteriophage P2 [132]. These two domains are linked by an oligonucleotide/oligosaccharide-binding (OB) feature located within the gp5 region [29, 133, 134].

Two categories of VgrG are associated with T6SSs, these being either the canonical or evolved VgrG types [135]. Canonical VgrG are thought to only play a mechanical or structural role in regard to the T6SS and consist of N–terminal gp27 domains and C-terminal gp5, beta strand repeat regions only [136–138]. Evolved VgrG feature additional domains and sequence extensions at their C-terminal ends; these regions can be involved in either effector-binding or in some cases be toxin domains themselves [35, 80, 139].

Domains known to be associated with evolved VgrG include the T6SS_Vgr domain as well as the Domain of Unknown Function, DUF2345 [122, 138, 140]. While the exact role of DUF2345 is not known, this VgrG region has been shown to be essential for T6SS assembly in *A. baumannii* [134]. In other species, DUF2345 may function as an effector chaperone [141, 142] or even be an effector domain itself. In *Klebsiella pneumoniae* DUF2345 has been shown to exert an antibacterial effect in competition experiments [52].

Within the genome sequence of *N. subflava* strain KU1003-01 are genes encoding seven full length ‘evolved’ VgrG that are >800 residues in length and three partial VgrG CDSs. According to Genomic SMART analysis [77], the full length VgrG have three main domains: an N-terminal GPD domain; a central Vgr / Rhs domain; and a C-terminal DUF2345 (COG4253). Analysis of the same sequences using Phyre^2^ [78] highlighted the presence of putative gp5 domains with oligosaccharide-binding (OB)-fold. T6SS-A type VgrG also feature extended C-terminal sequences following DUF2345 that vary in length and composition.

**Fig. 4.**
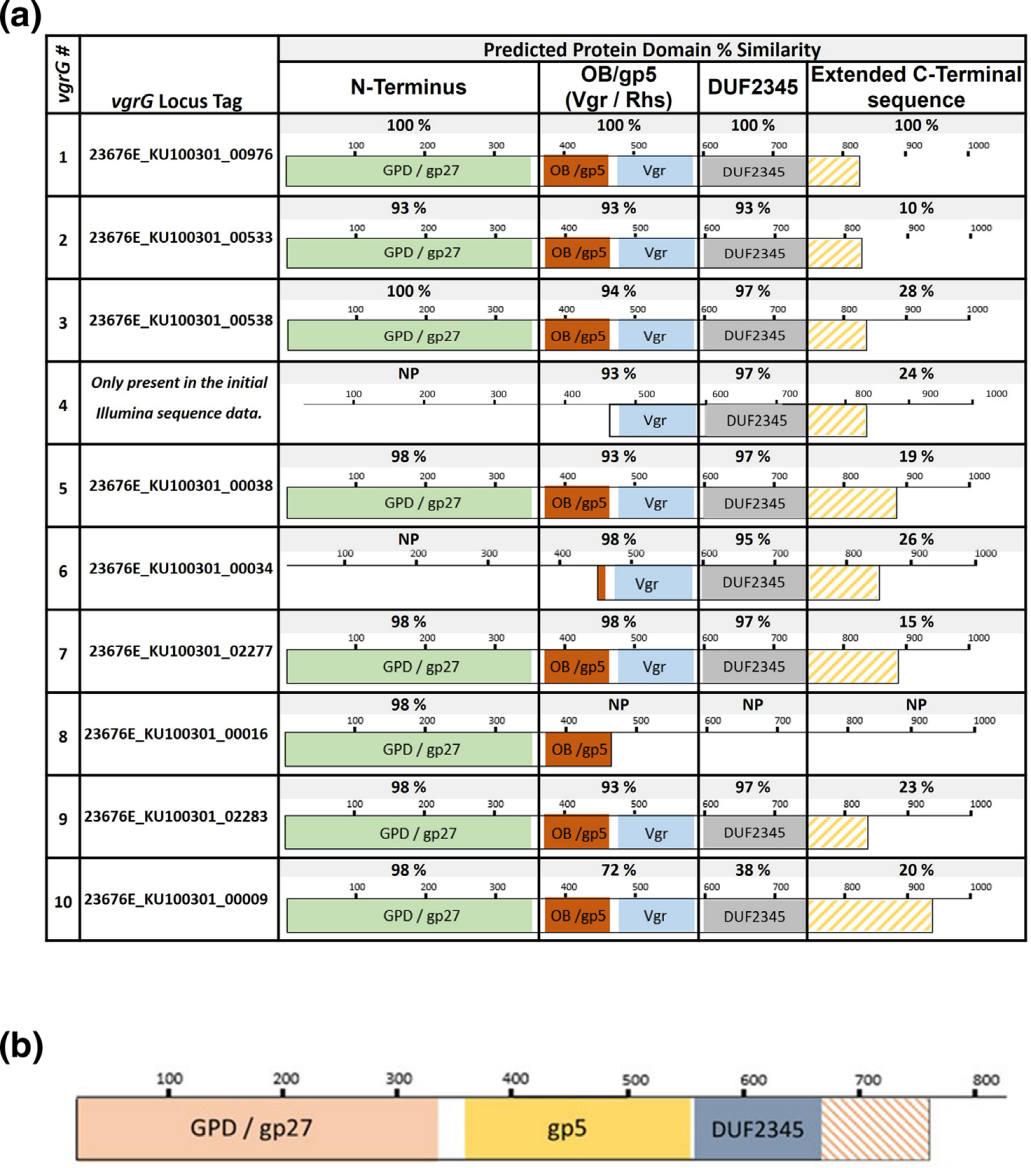
Protein domains of the *Neisseria* spp. T6SS VgrG. VgrG domains with structures similar to bacteriophage proteins. Phage hub proteins, GPD in P2 bacteriophage or gene product 27 (gp27) of bacteriophage T4. The oligonucleotide/oligosaccharide-binding (OB) domains resemble regions within the T4 gene product 5 (gp5) [29, 133]. T6SS_Vgr domains are associated with some VgrG types [122] and are followed by Domains of Unknown Function (DUF2345). (**a**) VgrG domains, predicted using Genomic SMART [77] and Phyre^2^ [78], as well as their approximate locations within VgrGs 1–10 of *N. subflava* strain KU1003-01. The percent similarity of each domain in comparison to the VgrG1 copy, which is encoded at the core cluster (see Fig. 1, location B) are shown highlighted in grey above each image. VgrG4, VgrG6, and VgrG8 each lack one or more domains seen in the complete Type VI Secretion System tip protein, VgrG. (**b**) The approximate domain locations predicted for the single VgrG present in the T6SS-B in *N. subflava* strains KU1003-02, RH3002-v2g and ATCC 49275. These VgrG are between 98–100 % similar to one another but only around 25 % similar overall to VgrG1 of *N. subflava* strain KU1003-01. The domains of the T6SS-B type VgrG are shown in different colours to the T6SS-A type to highlight these being different VgrG types.

The approximate domain locations for the VgrG of *N. subflava* strain KU1003-01 and their percentage similarity in comparison to VgrG1 encoded at the core cluster are shown in Fig. 4a. A high level of amino acid similarity can be seen across each of the domains with the exception of the extended C-terminal sequences which only share between 12 and 50% similarity to one another. The lowest percentage similarity scores can be seen for VgrG10, this is thought to be a specific VgrG sequence type and associated only with Rhs effector types in the T6SS-A *Neisseria* spp.

VgrG with specific C-terminal sequences identified in *N. subflava* strain KU1003-01 are also present in other *Neisseria* spp. and associated with homologous downstream genes (Table 2). This finding suggests that VgrG with specific C-terminal sequences are associated with certain effector types across *Neisseria* spp. These are similar to the ‘cargo’ type effectors described by [26].

**Table 2.** Putative T6SS effectors associated with *N. subflava* strain KU1003-01 *vgrG* 1–10 and their presence in the genome sequences of other *Neisseria* spp. isolates

* * N. subflava* strain KU1003-01		† Isolates with homologous VgrG C-terminals and downstream genes.		‡ VgrG C-terminal similarity to KU1003-01 (%)	§ Products of genes downstream from VgrG with homologous C- terminals.	¶ Product similarity to KU1003-01 (%)	# Predicted SMART domains	** Predicted function
VgrG	Extended C - terminal (AA)	Species	Strain					
**1**	**80**	*** N. subflava ***	***C2011020198***	**100**	**Hydrolase**	**96**	**PGAP-1**	**Alpha/Beta Hydrolase**
		*** N. subflava ***	26 870	**99**		**96**		
		*** N. subflava ***	43 794	**99**		**96**		
		*** N. subflava ***	44 615	**99**		**96**		
		*** N. subflava ***	50 940	**99**		**96**		
		*** N. subflava ***	82 623	**99**		**96**		
		*** N. perflava ***	***UMB0023***	**99**		**96**		
		*** N. perflava ***	***UMB0210***	**99**		**96**		
		*** N. zoodegmatis ***	***DSM 21643***	**86**		**62**		
		*** N. zoodegmatis ***	***NCTC12230***	**86**		**62**		
**2**	**105**	*** N. subflava ***	**C2011009653**	**100**	**LysM domain protein with hydrolase / Peptidase M15 domains (LysM1**)	**99**	**LysM, Hydrolase, Peptidase**	**Endopeptidase**
		*** N. subflava ***	44 620	**98**		**99**		
		*** N. mucosa ***	**CCH7-A10**	**99**		**88**		
		*** N. mucosa ***	**FDAARGOS_260**	**99**		**88**		
		*** N. mucosa ***	**ATCC 19696**	**99**		**88**		
–	**NP**	N. ***N. sicca / mucosa***	**C2014002478**	**NP**	**Peptidase M15 domain only - within polymorphic T6SS toxin loci**.	**90 % similarity to LysM1 C-terminal**.	**Peptidase**	**Endopeptidase (Phage Endolysin**)
		*** N. subflava ***	**C2011020198**					
		*** N. perflava ***	**UMB0023**					
		*** N. perflava ***	**UMB0210**					
		*** N. subflava ***	26 870					
		*** N. subflava ***	43 794					
		*** N. subflava ***	44 615					
		*** N. subflava ***	50 940					
		*** N. subflava ***	**ExNM715**					
		*** N. subflava ***	39 722					
**3**	**87**	*** N. subflava ***	43 741	**99**	**Hypothetical T6SS Effector Protein 1**	**99**	**nd**	**Putative T6SS Toxin Cassette with unknown function**.
–	**NP**	N. ***N. mucosa / sicca***	**ExNM702**	**NP**	**Partial hypothetical T6SS Effector Protein 1. Within polymorphic T6SS toxin loci**.	**85**		
		N. ***N. mucosa / sicca***	**ExNM703**			**85**		
		N. ***N. sicca strain***	**VK64**			**92**		
**4**	**109**	*** N. subflava ***	**M18660**	**100**	**LysM domain protein with Peptidase domain (LysM2**)	**93**	**LysM, Peptidase**	**Transpeptidase**.
		*** N. subflava ***	43 741	**100**		**93**		
**5**	**136**	*** N. elongata ***	**C2013010062**	**99**	**phospholipase D (PlD1**)	**94**	**Twin PlD active site motifs**	**Phospholipase**.
		*** N. elongata ***	**M15910**	**99**		**93**		
**6**	**100**	*** N. mucosa ***	**CCH7-A10**	**99**	**Nuclease**	**88**	**VRR-NUC**	**Nuclease / Hydrolase**.
		*** N. mucosa ***	**FDAARGOS_260**	**94**		**88**		
		*** N. mucosa ***	**ATCC 19696**	**99**		**88**		
		N. ***N. sicca / mucosa***	**UMB0321**	**98**		**88**		
**7**	**140**	*** N. subflava ***	44 620	**99**	**phospholipase D (PlD2**)	**97**	**Twin PlD Active site motifs**	**Phospholipase**.
		*** N. subflava ***	43 741	**99**		**91**		
		*** N. flavescens ***	**CD-NF2**	**99**		**92**		
		*** N. mucosa ***	**CCH7-A10**	**90**		**90**		
		*** N. mucosa ***	**FDAARGOS_260**	**90**		**85**		
		*** N. mucosa ***	21 061	**90**		**89**		
**8**	**GPD (gp27) domain only**	*** N. subflava ***	**ExNm709**	**NP**	**Homologue of *N. meningitidis* UmuC**	**88**	**UmuC**	**Unknown in relation to T6SS**.
		*** N. subflava ***	44 616			**89**		
		*** N. subflava ***	**NJ9703**			**88**		
		*** N. subflava ***	**C2008001664**			**86**		
		*** N. subflava ***	**ExNm715**			**88**		
–	–	*** N. subflava ***	38 979			**74**		
		*** N. subflava ***	42 060			**78**		
		*** N. subflava ***	43 793			**74**		
		*** N. subflava ***	49 571			**76**		
		*** N. subflava ***	49 367			**78**		
		*** N. subflava ***	82 624			**88**		
**9**	**85**	*** N. subflava ***	82 618	**99**	**Hypothetical T6SS Effector Protein 2 (within ecDNA sequence**).	**74**	**HD Motif**	**Putative Metal Dependant hydrolase**.
		N. ***N. mucosa / sicca***	**26577_GT4A_CT1**	**97**		**86**		
		*** N. mucosa ***	**CCH7-A10**	**96**		**84**		
		*** N. mucosa ***	**FDAARGOS_260**	**98**		**80**		
		N. ***N. sicca / mucosa***	**UMB0321**	**99**		**73**		
		*** N. elongata ***	**C2013010062**	**65**		**67**		
		*** N. elongata ***	**C2010010207**	**65**		**69**		
		*** N. elongata ***	**M15910**	**65**		**69**		
**10**	**168**	*** N. subflava ***	**C2011020198**	**100**	**Rearrangement hotspot (Rhs) gene**	**91**	**nd**	**Unknown**.
		*** N. perflava ***	**UMB0023**	**100**		**90**		
		*** N. perflava ***	**UMB0210**	**100**		**90**		
		*** N. subflava ***	50 940	**100**		**93**		
		*** N. subflava ***	26 870	**99**		**93**		
		*** N. subflava ***	44 620	**99**		**98**		
		*** N. mucosa ***	**FDAARGOS_260**	**100**		**90**		

*VgrG identification numbers for *N. subflava* strain KU1003-01 as well as the length of extended C-terminal sequences following DUF3245 for each.

†*Neisseria* spp. identified with homologous C-terminal VgrG sequences.

‡VgrG C-terminal sequence similarities in comparison to *N. subflava* strain KU1003-01.

§Gene products predicted downstream from VgrG with homologous C-terminal sequences as well as their percent similarities.

¶To those in *N. subflava* strain KU1003-01.

#Domains predicted by (SMART) webserver, version 9.0 [77] for gene products downstream of VgrG, as well as predicted protein function (**).

nd Domains not detected or could not be confidently predicted. The gene products downstream of VgrG2 and VgrG3 are also present within T6SS polymorphic loci but are encoded away from VgrG. The gene product downstream of VgrG8 in *N. subflava* strain KU1003-01 is not encoded downstream of VgrG for some strains, although in others this is encoded next to truncated VgrG with no C-terminal sequences. (-) either VgrG or C-terminal sequences are absent. (NP) VgrG sequences are not present and could not be compared.

While the T6SS-B type VgrG also have three main domains; an N-terminal GPD domain followed by a gp5 like domain containing internal repeats and a short DUF2345, T6SS-B type VgrG do not feature diverse C-terminal regions (Fig. 4b). The single VgrGs encoded within the genomes of T6SS-B *N. subflava* strain KU1003-02, *N. subflava* strain RH3002-v2g, and *N. subflava* strain ATCC 49275 are around 99 % similar to one another along their entire lengths and only share around 25 % similarity overall to the T6SS-A type VgrG. No T6SS-A type VgrG could be identified in the genome sequences of the T6SS-B only species.

### T6SS effectors in *N. subflava* strain KU1003-01

**Fig. 5.**
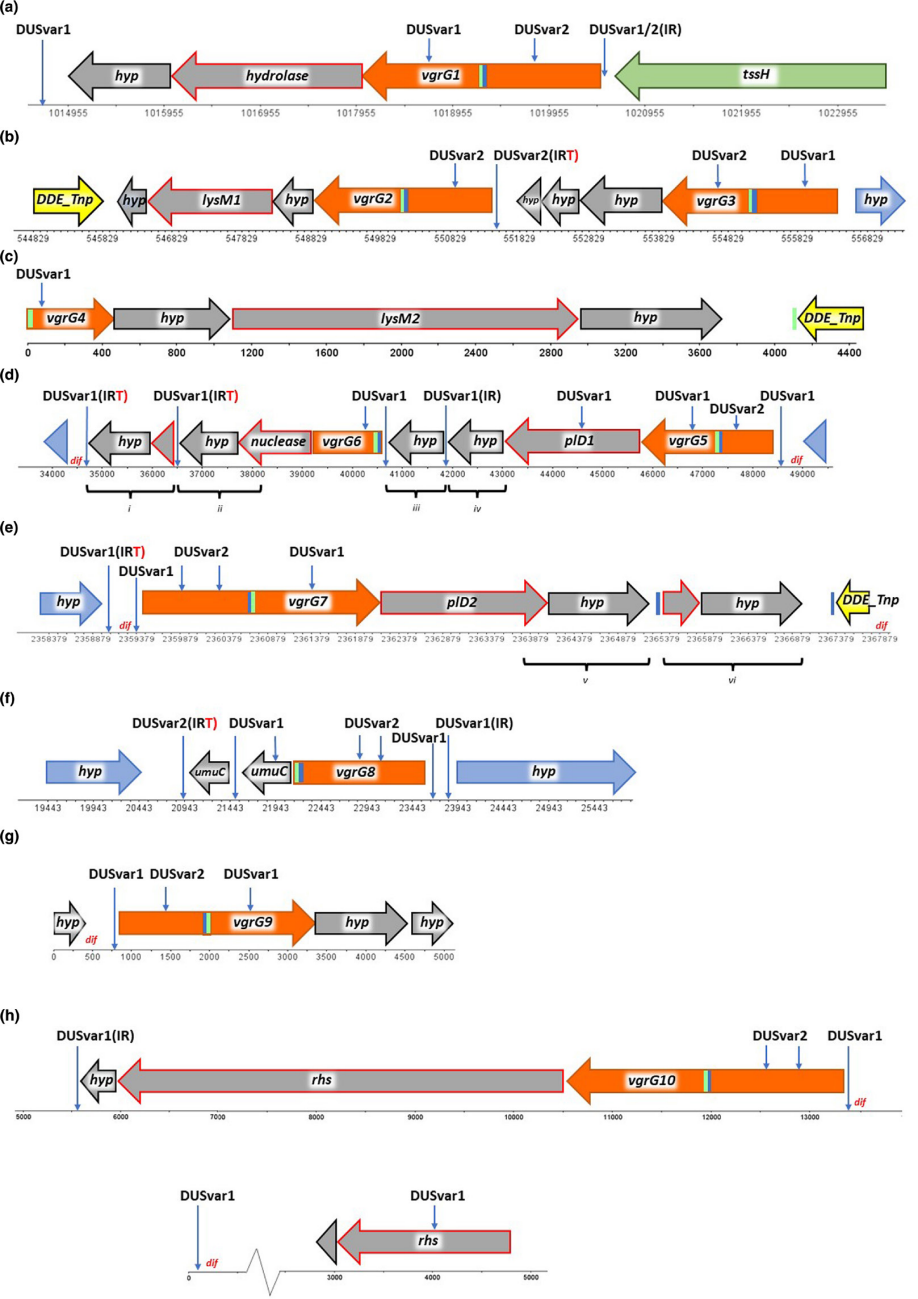
*
N. subflava* strain KU1003-01 *vgrG* 1–10 and downstream EI gene clusters. The *vgrG* (orange), putative T6SS effectors (grey with a red border), hypothetical genes (hyp) downstream of *vgrG* and putative T6SS effectors (grey with a black border), non-T6SS genes (blue), transposases (yellow), and short sequences (vertical green and blue bars highlighting sequences within both the N-terminal Gp5 OB fold regions of vgrG as well as homologous sequences located outside of the vgrG CDS) were analysed. In particular, locations of DNA Uptake Sequence variants (DUSvar), Inverted repeats (IR), Inverted repeats predicted to be transcriptional terminators (IRT), and *dif* recombinase sequences (*dif*) where all present associated with *vgrG* regions. Duplications were identified associated with *vgrG* 6 (d, i and ii, iii and iv) and *vgrG* 7 (e, v and vi). The *vgrG* 9 copy (**g**) is predicted to be on a short, circular sequence, based on data from both combined MinION/Illumina enhanced genome sequencing.

Table 2 highlights the putative T6SS effectors encoded downstream of each *vgrG* for *N. subflava* strain KU1003-01 as well as homologues identified in other *Neisseria* spp. The predicted effectors include proteins with LysM, hydrolase and peptidase domains, two phospholipase D variants, a nuclease, and Rhs. The organisation of *vgrG*, downstream effector (E), and hypothetical genes thought to encode immunity proteins (I) are shown in Fig. 5 (a–h). Additional features of these gene clusters include *dif* recombination sites and neisserial DNA Uptake Sequences (DUS). The vertical blue and green bars highlight short sequences within both the N-terminal Gp5 OB fold regions of *vgrG* as well as homologous sequences located outside of the *vgrG* CDS.

### Additional features of the *vgrG* EI gene clusters

In regard to the *dif* sites identified in *N. subflava* strain KU1003-01, these flank the *vgrG*5/6 (Fig. 4d) and *vgrG*7 (Fig. 5g) clusters. A single *dif* site is also present at one end of the *vgrG*9 cluster (Fig. 5e). It is possible these gene clusters exist as either one or several genomic islands, flanked by *dif*, similar to those flanking the horizontally acquired gonococcal genomic island (GGI) in most *N. gonorrhoeae* strains [143] and a significant number of serogroup C, W-135, and X *N. meningitidis* isolates [144]. In *N. gonorrhoeae *, the GGI has been shown to excise from the chromosome at *dif* sites and exist as transient extrachromosomal circular DNA following its excision [145]. The genomic region where *dif* are present is shown on Fig. 1 and marked ‘*dif*’.

In the pathogens, *dif* sequences are targeted by the site-specific recombinase XerCD. In *N. gonorrhoeae dif* consists of the 28 bp sequence; AGTTCGCATAATGATATTATGTTAAAT and in *N. meningitidis *; AGTTCGCATAATATATATTATGTTAAAT [145, 146]. While the *dif* sites in *N. subflava* strain KU1003-01 have identical XerD recognition binding sequences to those in the pathogens as well as core sequences that differ by only one base, the XerC binding sequences are different to those in the pathogens. *N. subflava* strain KU1003-01 has two XerC binding sequence variants these being either; AAACTACATAA or AAGGAAAATAA.

Complete 12 mer and shorter 11 mer copies of neisserial DUS variants are present both within the CDS of *vgrG*, as well as the coding and non-coding regions both up and downstream from *vgrG* in *N. subflava* strain KU1003-01. While DUSvar1 [147] are the overall dominant type in *N. subflava* strain KU1003-01 [64] as well as other *N. subflava* biovar *flavescens*, and *N. elongata* [147], and DUSvar2 are the dominant type within *N. mucosa* and *N. sicca* [147], both DUS types, DUSvar1 and DUSvar2, are present in and around *vgrG* of *N. subflava* strain KU1003-01.

The presence of DUS in and around genes that may provide a competitive advantage could explain conservation and sharing of these genes across the commensals. While *Neisseria* spp. are naturally competent [148, 149] and preferentially take up DNA containing DUS [150], DUS are also known to regulate gene expression [151] and a quarter of all neisserial genes are thought to be either attenuated or terminated by IR sequences [129].

In regard to DUS IRs that act as Rho independent transcriptional terminators, these have spacer sequences in between the repeats that are smaller than or equal to 20 nucleotides in length [129] as well as Thymine (T) rich regions following the IR repeat [152]. A number of DUS (IRs) within the *vgrG* EI clusters of *N. subflava* strain KU1003-01 fit the criteria for being transcriptional terminators as defined by Ambur, Frye and Tønjum, [129]. The DUS IRs predicted to be Rho-independent terminators using ARNold [130] for each of the *vgrG* clusters are highlighted on Fig. 5 with T.

### Gene duplications within the *vgrG* EI clusters

Regions i, ii, iii, iv, v and vi, highlighted on Figs 5d, e represent duplications within the *vgrG*5*, vgrG*6 *and vgrG*7 clusters. In regard to the *vgrG*6 cluster, the four genes downstream of *vgrG* are arranged in pairs, the two genes following *vgrG*6 are thought to encode the EI pair. The first, shorter ‘gene’ of the second pair within region i encodes a protein that is 97 % similar to the C-terminal region of the nuclease encoded within region ii. This partial CDS is followed by a gene encoding a product with DUF3396 that is 85 % similar to the gene product within region ii. Genes encoding DUF3396 have been identified in *Acinetobacter* spp. and downstream from T6SS associated nucleases by Repizo *et al*. [122]. These are thought to encode nuclease immunity proteins [153].

Regions iii and iv (Fig. 5d) represent a duplication of a putative phospholipase D (PlD) immunity gene within the *vgrG*5 cluster. The gene products of iii and iv are 94 % similar to one another. Using a WU-blast 2.0 search against SecReT6 v2.0 database both were identified as having similarities to PlD immunity protein PA5087 of *Pseudomonas* spp. [154] with an E-value of 3.5×10^−12^. Regions v and vi (Fig. 5e) represent duplications within the *vgrG*7 cluster. The gene pair following *vgrG*7 are believed to encode the EI pair. Similar to the arrangement of genes for the *vgrG*6 cluster, the first and shortest sequence within the second gene pair (region vi) is a partial gene encoding a product that is 79 % similar to the C-terminal region of the PlD within region v. Following this partial gene is a CDS whose product is 88 % similar to the putative PlD immunity gene. Using a WU-blast 2.0 search against SecReT6 v2.0 database both putative PlD protein sequences were identified as having similarities to PlD immunity protein PA5087 of *Pseudomonas* spp. [154] with E-values of 4.0×10^−20^ and 1.7×10^−24^.

While T6SS poly-Immunity loci can exist and are associated with T6SS, in some species there is variation in the number and type of immunity genes they possess [155–157]. In the case of nuclease effectors, these are frequently identified along with a diverse array of immunity genes [157]. While duplication is the most likely explanation for more than one immunity gene present at *vgrG* loci, the duplications may not have originated in *N. subflava* strain KU1003-01, this being due to identical gene organisations being found in genome sequences of *N. mucosa* and *N. elongata *.

## Conclusions

Our analyses reveal that T6SS have been long established in the *Neisseria* spp. While there is evidence to support HGT of T6SS-B core genes between non-pathogenic *Neisseria* spp., this is not the case for the T6SS-A core gene clusters. There is additional evidence to support sharing of the T6SS-A *vgrG* and EI gene clusters identified in *N. subflava* strain KU1003-01. The presence of homologues in a number of other genome sequences of *N. subflava* biovar *perflava / flavescens*, *N. mucosa *, *N. sicca,* and *N. elongata* supports T6SS-A *vgrG* and EI HGT. Our analyses suggest that a diverse range of putative T6SS toxins and immunity genes may exist for the two T6SS types and subtype in *Neisseria* spp. A full investigation into their true diversity needs to be conducted. While *N. subflava* is usually documented as a commensal, in some literature this species has been referred to as an opportunistic pathogen making investigations into its T6SS and any potential role in disease important in its own right. T6SS elements are not present in the human pathogens, *N. gonorrhoeae* and *N. meningitidis *. This may suggest that the commensals utilise their T6SS for niche competition. Characterising neisserial T6SS effectors may therefore provide an avenue for the future development of novel therapeutic options in the treatment of multidrug resistant infections.

## Supplementary Data

Supplementary material 1
